# The Trail Pheromone of the Venomous Samsum Ant, *Pachycondyla sennaarensis*

**DOI:** 10.1673/031.011.0131

**Published:** 2011-03-18

**Authors:** Ashraf Mohamed Ali Mashaly, Ashraf Mohamed Ahmed, Mosa Abdullah Al—Abdullah, Mohamed Saleh Al—Khalifa

**Affiliations:** ^1^ Department of Zoology, College of Science, King Saud University, PO. Box: 2455, Riyadh, 11451, Kingdom of Saudi Arabia; ^2^ Department of Zoology, Faculty of Science, Minia University, El Minia, Egypt

**Keywords:** Dufour gland, longevity, optimal dose, source, specificity

## Abstract

Ant species use branching networks of pheromone trails for orientation between nest and resources. The current study demonstrated that workers of the venomous samsum ant, *Pachycondyla sennaarensis* (Mayr) (Hymenoptera: Formicidae: Ponerinae), employ recruitment trail pheromones discharged from the Dufour's gland. Secretions of other abdomen complex glands, as well as hindgut gland secretions, did not evoke trail following. The optimum concentration of trail pheromone was found to be 0.1 gland equivalent/40 cm trail. This concentration demonstrated effective longevity for about one hour. This study also showed that *P. sennaarensis* and *Tapinoma simrothi* each respond to the trail pheromones of the other species as well as their own.

## Introduction

The study of insect pheromones has demonstrated their pivotal roles in social organization, recognition, mate choice, aggregation, and territoriality (Wyatt 2003). Pheromone trails illustrate how insect behavior is modulated by pheromones. Foraging insects use pheromone trails to lead colony members to food sources and new nest locations (Horn 1976). In trail communication, already excited individuals are led along a trail to the target area (Hölldobler and Wilson 1990; Traniello and Robson 1995). This pheromonal communication system is based on the release of chemicals from a variety of specialized glands that then form a trail (Morgan 1990).

In ants, different recruitment mechanisms include tandem running in which the scout ant leads one nest mate to the resource, group recruitment which recruits tens of nest mates, and mass communication which uses pheromones to recruit large numbers of nest mates (Wyatt 2003). Which recruitment mechanism is used depends as much on the ecology of the species as its taxonomic position. Comparing closely related species with different foraging strategies reveals that key factors that select for recruitment behavior are clumped, patchy food resources (Traniello and Robson 1995). For example, the ant *Pachycondyla obscuricornis* hunts small arthropods, and it has no need for foraging recruitment as each prey item can be carried back to the nest by the finder. However, for nest moving this species does use tandem running, facilitated by pygidial gland pheromones (Hölldobler et al. 1978; Kugler 1978). In the tandem running recruitment technique in the ponerine ant, *P. tesserinoda,* Maschwitz et al. (1974) and Hölldobler and Traniello (1980) discovered that both a surface pheromone and tactile stimuli permit the formation of a communication bond between the two individuals of a tandem running pair.

The samsum ant, *Pachycondyla sennaarensis* (Mayr) (Hymenoptera: Formicidae: Ponerinae), is the most common ant in savannah regions of Sudan (Levieux and Diomande 1978). It has been recorded in Saudi Arabia (Collingwood 1985), Kuwait, Oman, and Yemen (Collingwood and Agosti 1996), in the United Arab Emirates (Collingwood et al. 1997), in Iran (Akbarzadeh et al. 2004; Tirgari and Paknia 2005) as well as many locations in Africa (Taylor 2005). In Saudi Arabia it has established itself in both urban and rural areas and is closely related to the human activity sites. The ants seemed to have adapted to the hot and dry weather of Riyadh Region especially in spring and summer by establishing the nests in moist sand in irrigated gardens, parks, housing areas and roadside plantations (Al-Khalifa et al. 2010). Because of their ability to sting, these ants have medical importance (Tirgari et al. 2004) and are considered as a significant public health hazard in Saudi Arabia (Al-Anazi et al. 2009). It has been described as unique among ponerines in its seed-eating habits (Lachaud and Dejean 1994).

This study was conducted for three reasons: first, the widespread distribution of this species in Saudi Arabia; secondly, its threat as a health problem in that it may cause anaphylaxis in sensitive persons and even lead to death (Al-Shahwan et al. 2006); and third, trail pheromones have never been investigated in this species. The current study investigates the role of trail pheromone in the behavior of *P. sennaarensis,* including the glandular origin, optimal concentration, longevity, and specificity of the trail pheromone.

## Materials and Methods

### Insects

Colonies of *P. sennaarensis* (containing 1500–2500 workers, with brood of all stages and multiple queens (5–10), were collected from Al Ehsaa Governorate, East Riyadh, and the Kingdom of Saudi Arabia. Collected nests were moved to the ant insectary in the Zoology Department, College of Sciences, King Saud University. Ants were housed in plastic nest-bottles within a large plastic box (45 × 30 × 18 cm) that was used as a foraging area. Fluon-coated walls prevented ant escape. The insectary was maintained at 28 ± 1° C, ∼ 30% RH, and 12:12 L:D. Ants were allowed to access fresh water and sugar syrup in glass tubes blocked with cotton wool, fed daily with mealworm larvae, and weekly with apple sauce. Ten days prior to the start of the experiment and during the experiment, ants were not given sugar sources to ensure that they would readily form foraging trails to the sugar syrup feeder. To prevent drying up nests were moistened by adding a few drops of water when needed.

### Trail pheromone experiments

The determination of the origin of the trail pheromones involved bioassay studies in which suspensions of the potential organ sources were presented to the ants via artificial trails. The following organs were tested: pygidial, poison, Dufour's glands, and hindgut. Experiments were carried out as described in Hölldobler and Wilson (1970) and Hölldobler (1976). Three experimental groups (each ≈500 workers) were housed in sand-filled Plexiglass containers (45 cm × 30 cm × 18 cm). Each of these replications (≈500) workers originated from a different colony.

Each nest was connected to a foraging arena with a sand-covered floor (2 cm thick). The ants were allowed to reach the arena by means of two cardboard bridges (each with 1cm wide and 40cm long). The bridges originated close to the nest entrance hole and diverged at approximately 30°, thus forming a V-shaped double bridge that connected to the arena at two points approximately 15 cm apart. Source glands were ground in a small glass tissue grinder in 100 µl hexane and were drawn along one branch of the V-bridge, using a 0.8 mm Standard graph pen (Standardgraph Zeichentechnik GmbH, www.standardgraph.de). As control, equivalent amounts of hexane were used as a trail on the other side of the V-bridge. After the solvent had evaporated (30 seconds post-application), both test and control trails were placed simultaneously at the ant nest entrance. At the end of the test, ants that entered the arena through the openings of the treated and control bridges were counted. Each test was conducted five times and the activity was calculated as the mean number of responding ants. Each V-bridge was used only once in an experiment and at least 30 min elapsed between subsequent trail tests, but in each case the test group was tested only once each day. After each test all ants were returned back to their respective nests. Before and after each test, the Standard graph pen was cleaned thoroughly with hexane. The last washing was used in a blank trail test to ensure there was no residual activity from the last test. As a negative control, a drawn circle made with a pencil on the paper (with no chemical trail laid) was also presented to the ants to ensure that it no effect on the results.

This experiment was conducted to determine whether combinations of gland extracts were synergistic (Mashaly 2010). Thus, a mixture of extracts of the Dufour's gland and the pygidial gland, Dufour's gland, the poison gland and Dufour's gland, and Dufour's gland and the hindgut suspended in 100 µl hexane were used. Additionally, a whole abdomen extract in 100 µl hexane was applied in the same way. Worker ants were allowed to access the treated foraging area for 20 minutes after which ants within the foraging area were counted. This test was conducted five times and the mean number of responding ants was calculated and compared with that that of control experiment (hexane treated areas).

The optimal concentration of the trail pheromone was determined using eight concentrations of the source gland (0.001, 0.01, 0.1, 1, 5, 10, 20, and 40 gland equivalent (GE) per 40 cm trail) in 100 µl hexane. Worker ants were allowed to access the foraging area for 20 min. Each test (for each individual concentration) was conducted five times for test and control experiments. The mean number of responding ants was calculated for each concentration and compared to the number of ants that that responded to hexane alone (control).

To determine the longevity of trail pheromones, the optimal concentration obtained from the previous experiment was applied as before and ants were allowed access to the foraging area at different time periods post-application including 0, 15, 30, 45, and 60 min. Ants were allowed 20 min to access the foraging area. This test was conducted five times and the mean number of responding ants was calculated and compared to the number of ants that responded to hexane alone for each time period.

To test the specificity of trail pheromones between the samsum ant and *Tapinoma simrothi* Krausse-Heldrungen (Hymenoptera: Formicidae: Dolichoderinae), the hexane extract of the source gland of each species was tested against the other species using the method described above. This test was conducted five times and the mean number of responding ants was calculated.

All statistical analyses were undertaken using MINITAB software (MINITAB, State College, PA, Version 13.1, 2002). Data from all experiments were first tested for normality using Anderson Darling test, and for homogeneity of variance prior to any further statistical analysis. Because data were not normally distributed, Kruskal-Wallis was used to test the overall differences prior to individual comparisons within treatments using the Man-Whitney non-parametric *U* test.

## Results

Single scouts of *P. sennaarensis* leading nestmates by tandem running to newly discovered food sources was observed, but the number of ants recruited in this way was too small in all of the experiments to demonstrate a significant recruitment effect. Only 1–4 tandems/h were recorded at the food source in this experiment.

**Figure 1.**
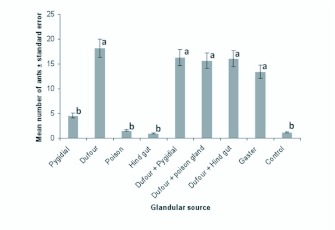
Number of *Pachycondyla sennaarensis* workers recruited by hexane extracts of, whole abdomen, hindgut contents, pygidial, or Dufour's gland secretions. Hexane was used as the control. Error bars represent the standard errors of the mean of five replicates. * Significant difference compared to control. High quality figures are available online.

In the preliminary tests, the ability of different abdominal glands (Dufour's, pygidial, and poison glands) and hindgut to elicit trail following was tested against a hexane control. Since ants followed the each of the abdominal gland extracts over hexane in all cases, the various abdominal glands were tested against each other (Figure 1). Dufour's gland showed significantly higher activity when compared to the pygidial gland, poison gland or hindgut (18.2 ± 1.4 ants *v* 3.6 ± 0.5, 1.61 ± 0.34 and 1.01 ± 0.4 respectively) (*P* < 0.05, n = 5, Mann Whitney *U* test) (Figure 1). Dufour's gland secretion was also more active than whole abdomen extracts (18.2 ± 1.4 *v* 13.4 ± 1.5) (*P* < 0.05, n = 5, Mann Whitney *U test*) (Figure 1). The pygidial gland, poison gland, and hindgut induced activity similar to that of the control 3.6 ± 0.5, 1.61 ± 0.34, and 1.01 ± 0.4 *v* 1.25 ± 0.3 respectively) (*P* > 0.05, n = 5, Mann Whitney *U* test).

To assess whether more than one gland was involved in trail signaling, combined extracts of Dufour's gland and poison glands, Dufour's gland and pygidial glands, as well as Dufour's gland and hindgut were compared to that of the Dufour's gland alone. The results revealed no significant difference between the activity induced by the Dufour's gland alone and that induced by the combined extracts or that of the abdomen alone (*P* < 0.05, n = 5, Mann Whitney *U* test). This indicates that the Dufour's gland secretion accounts for all the trail pheromone activity, and that there is no synergism when combined with other sources (Figure 1).

**Figure 2.**
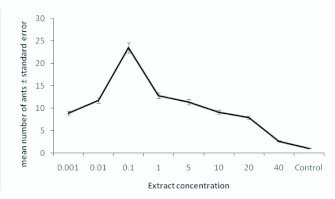
Number of *Pachycondyla sennaarensis* workers responding to an artificial trail of different concentrations of whole abdomen extract. Error bars represent the standard errors of the mean of five replicates. High quality figures are available online.

Eight concentrations of whole abdomen extract were tested (0.001, 0.01, 0.1, 1, 5, 10, 20, and 40 equivalents in 100µl hexane per 40cm trail) to determine the optimal dose. The highest response of worker ants was evoked at 0.1 GE/40 cm trail. This activity significantly decreased at concentrations below and above this concentration optimal (*P* < 0.05, n = 5, Mann Whitney *U* test) (Figure 2).

**Figure 3.**
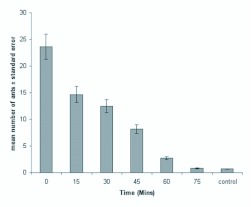
Longevity of the trail pheromone of *Pachycondyla sennaarensis.* Error bars represent the standard errors of the mean of five replicates. High quality figures are available online.

Pheromone trail longevity was tested for by allowing worker ants 20 min access at varying lengths of time (0, 15, 30, 45, and 60 min) after applications of the optimal concentration (0.1 GE/40 cm trail) of whole abdomen extract. As shown in Figure 3, the activity of trail pheromone decreased dramatically within one hour.

Specificity of trail pheromones of *P. sennaarensis* and *T. simrothi* was tested reciprocally by exposing workers of each ant species to the trail pheromone of the other. Both species responded to the trail pheromone of the other (Table 1).

## Discussion

In this study, various aspects of the trail pheromone of the samsum ant, *P. sennaarensis,* were investigated, including the source, the longevity, the optimal effective concentration and interspecific responses. A better understanding the biological aspects of this venomous ant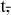 may help in producing useful control measures in the future.

In ants, a diversity of recruitment strategies, including tandem running, group recruitment, and mass recruitment are described. From an evolutionary perspective, tandem running appears to represent the most primitive recruitment strategy (Hölldobler and Wilson 1990; Liefke et al. 2001). During tandem running, a scout that has discovered a food source leads a single nest-mate to the food (Möglich et al. 1974; Möglich 1979; Liefke et al. 2001). The nest mate keeps close antennal contact with the scout and only one nest mate is recruited per trip. The scout then performs an invitation display inside the nest. Invitation behavior may be accomplished by antennation, by the presentation of food samples, by agitated displays by the recruiter, or by secretion of chemicals that alert nestmates to the presence of a chemical trail that leads to the food finding. Ponerine ants are known to use visual cues and tandem running as orienting systems, while species using trail pheromone are far less common. Trail strategy in ponerine ants seems to occur in three contexts: foraging on immobile and large quantities of resources, species with army-ant-like behavior, and colony migration (Blatrix et al. 2002).

**Table 1.**
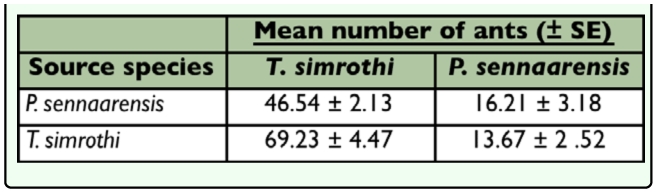
Interspecific responses to trail pheromones of *Pachycondyla sennaarensis* and *Tapinoma simrothi.* SE: standard errors of means of five replicates.

Our findings demonstrate that *P. sennaarensis* may be using a tandem running technique accomplished by a trail pheromone secretion from the Dufour's gland. This gland was also shown to be the source of trails deposited by several other ants; *Gnamptogenys menadensis, Ectotomma ruidum,* and *Gnamptogenys striatula* (Bestmann et al. 1995; Gobin et al. 1998; Blatrix et al. 2002). On the other hand, some other species belonging to the subfamily Ponerinae use poison and pygidial gland secretion for trail communication (Billen and Morgan 1998). In the subfamily Ponerinae, at least 3 genera have been characterized as having different types of sternal glands that serve in chemical trail communication. These are *Onychomyrmex* (Hölldobler et al. 1982), *Mystrium* (Hölldobler et al. 1998), and *Pachycondyla* (*Paltothyreus*) (Hölldobler 1984). Within the genus *Pachycondyla, P. tarsatus* is the only species known to possess distinct sternal glands that are employed in chemical trail communication (Janssen et al. 1999). All other studied species of *Pachycondyla* either recruit by tandem running, or by chemical trail communication using secretions from the pygidial gland. For example, citronellal has been identified as a trail pheromone in *P. marginata* (Hölldobler et al. 1996). Dufour's gland is the source of trail pheromones in many other genera, such as in *Monomorium pharaonis* (Hölldobler 1973), *M. mayri* (Mashaly 2010), *Solenopsis* species (Robert et al. 1989), *Pheidole fallax* Mayr (Law et al. 1965), and *Polyergus rufescens* (Visicchio et al. 2001). However, none of the other abdominal glands showed significant activity in these insects.

Our results did not indicate any synergism in trail following between the Dufour's gland and the pygidial, poison glands, or hindgut. Similar data has been obtained in three *Monomorium* ant species (Mashaly 2010), in *M. lepineyi* (Mashaly et al. 2010), and in cases of *Ph. jordanica* and *Ph. sinaitica* (Ali and Mashaly 1997).

It is well established that a specific concentration of trail pheromones is important since concentrations that are too high or too low elicit either no response or repellency (Barlin et al. 1976). Our results demonstrated that, the optimal concentration of the trail pheromone from *P. sennaarensis* was 0.1 GE/40 cm trail as previously found in *Iridomyrmex humilis* Mayr, where the optimal activity was in response to a trail containing of 0.1 – 1.0 ant GE/50 cm. The fact that activity dropped when the concentration was lower or higher than the optimal concentration is supported by the finding of Van Vorhis Key et al. (1981) in which the highest activity in *Tetramorium impurum* was reported as 0.1 poison GE/ 30 cm trail. The activity decreased at a concentration of 1.0 and 0.01 poison GE/ 30 cm trail, and totally disappeared at a concentration of 0.001 GE/ 30 cm trail. This is supported by the findings of Morgan et al. (1990). In *M. niloticum,* the optimum concentration was 1.0 and 0.1 whole abdomen GE/30 cm trail (Mashaly 2010).

Basically, pheromones are released mainly from exocrine glands as liquids that evaporate into the surrounding air (Bossert and Wilson 1963). The—distance through which a pheromone may transmit a message is a function of the volatility of the compound, its chemical stability in air, the rate of diffusion, olfactory efficiency of the receiver, and wind speed and direction (Fitzgerald and Underwood 1998). In ants, trail longevity varies from minutes in *Aphaenogaster albisetosus* (Hölldobler et al. 1995) to several weeks in some *Eciton* species (Torgerson and Akre 1970). Short-lived trails can rapidly modulate recruitment to ephemeral food sources, whereas long-lived trails will be more suited to persistent, or recurrent, food sources (Fitzgerald and Underwood 1998). Data of the current study showed that the activity of the optimal dose trail pheromone in *P. sennaarensis* decreased to half of the original activity level after about 30 min and completely disappeared after 1h. Similarly, in *Pheidole teneriffana,* the optimal dose of the trail completely disappeared after 1 h (Ali 1996). Longevity of a trail was found vary considerably depending on the ant species. For example, it was 75–90 min in *Ph. jordanica, Ph. Sinaitica,* and *Pheidole* sp. (Ali and Mashaly 1997), 2.5 hr in *M. pharaonis* Linnaeus (Blum 1966), 2h in *M. lepineyi* and *M. bicolor* (Mashaly et al. 2010), and 1h in *M. niloticum, M. mayri,* and *M. najrane* (Mashaly 2010).

Data from the cross-attraction experiment between *P. sennaarensis* and the related species *T. simrothi* suggests that they may be using the same chemicals in their trail pheromone. Evidence of this is shown by Blum and Ross (1965) who stated that *Tetramorium guineense* (Fabr.) trails were well followed by two species not closely related, *Atta texana* and *Trachmyrnex septentrionalis,* and that, they follow each other's artificial trail (Blum and Portocarrero 1966). Moreover, a trail laid with the poison gland of *Daceton armigeirm* was not followed by the workers of *Sericomyrmex urichi* and *D.* *armigeirm* itself, but it was strongly followed by *A. texana, A. cephalotes, A. sexdens, Trachmyrnex septentrionalis* and *Acromyrnmx octospinosus* (Robinson et al. 1974). *Tetramorium caespitum* and *Myrmica ruginodis* would follow the artificial trail from each other (Attygalle and Morgan 1984).

In conclusion, short lasting trail pheromones are secreted from the Dufour's gland in the samsum ant *P. sennaarensis.* Also, the concentration of the pheromone had a strong effect on worker behavior.
